# Analyzing public responses to Shanghai's strict smoking ban: A data mining study based on 12320 Health Hotline calls

**DOI:** 10.18332/tid/218152

**Published:** 2026-06-27

**Authors:** Lili Shi, Aksara Regmi, Jiaqi Shao, Zi-an He, Yuyang Cai

**Affiliations:** 1 Xinhua Hospital, Shanghai Jiao Tong University School of Medicine, Shanghai, China; 2 School of Public Health, Shanghai Jiao Tong University School of Medicine, Shanghai, China; 3 China Institute for Urban Governance, Shanghai Jiao Tong University, Shanghai, China

**Keywords:** smoking ban, hotline, natural language processing (NLP), tobacco control, social supervision

## Abstract

**INTRODUCTION:**

This study evaluates the public response to Shanghai's strict smoking ban through an analysis of calls made to the 12320 Health Hotline. The focus is on identifying the types of violations reported, the locations involved, and how these calls can inform future enforcement strategies.

**METHODS:**

We collected 41614 call records from March 2016 to March 2019 and used natural language processing (NLP) techniques to analyze these data. Calls containing tobacco-related complaints, consultations, help-seeking requests, or suggestions were included.

**RESULTS:**

Following the implementation of the amended regulations, tobacco-related hotline activity peaked in March 2017. During this month, 6937 eligible calls were recorded, of which 4705 (68%) were complaints, 1212 (17%) were consultations, and 829 (12%) were help-seeking requests, while only 172 calls (2%) involved suggestions. Workplaces accounted for the largest proportion of reported locations (35%; n=2399), followed by food and beverage establishments (14%; n=960) and leisure or hospitality venues (13%; n=902), with schools least frequently reported (1%; n=86).

**CONCLUSIONS:**

Hotline-based surveillance provides a practical approach for characterizing public concerns related to smoking ban enforcement. Further studies are needed to examine demographic predictors of call patterns, assess long-term trends beyond the initial implementation period, and explore how such data sources may complement conventional tobacco control monitoring systems.

## INTRODUCTION

China is the world’s largest producer and consumer of tobacco^1^, accounting for approximately 44% of global cigarette consumption^2^. In 2009, cigarette production in China reached 2.3 trillion units, representing nearly 40% of the world’s total output^1^. According to the 2018 Global Progress Report on the Implementation of the WHO Framework Convention on Tobacco Control (WHO FCTC), tobacco use was responsible for an estimated 1.59 million deaths in China^3^. Without comprehensive implementation and enforcement of effective tobacco control measures, global smoking prevalence is projected to increase to 22.0% by 2030^4^, and the annual number of tobacco-related deaths worldwide is expected to reach 8 million, more than 80% of which will occur in low- and middle-income countries^5^.

To address this substantial public health burden, the World Health Organization Framework Convention on Tobacco Control (WHO FCTC) was negotiated and adopted as the first global public health treaty aimed at reducing tobacco use and exposure to secondhand smoke. Ratified by 168 Parties, including China, the Convention was adopted by the World Health Assembly in May 2003 and entered into force in February 2005^6^. It requires Parties to enact and enforce comprehensive smoke-free legislation covering indoor public spaces, workplaces, and public transportation systems^7^.

Following ratification, China implemented a series of city-level smoke-free initiatives between 2004 and 2014. However, these early policies were largely characterized by partial indoor smoking restrictions and inconsistent enforcement, limiting their overall effectiveness^8^. In response to these challenges, the Chinese government introduced the Healthy China 2030 Plan Outline in 2016, which prioritized the full implementation of WHO FCTC measures nationwide^3^. This policy shift marked a new phase in China’s tobacco control strategy and was exemplified by Shanghai’s transition from a partial indoor smoking ban enacted in 2010 to a comprehensive smoke-free regulation implemented in 2017.

Evidence from multiple countries has demonstrated the effectiveness of smoke-free legislation in reducing smoking prevalence and improving population health outcomes. For example, research by Pieroni et al.^9^ found that individuals with a higher level of education tend to respond more strongly to smoking bans at both individual and collective levels, although the overall effects were modest^10^. Similarly, studies from Australia have shown that stricter smoke-free legislation is associated with increased quit probabilities, particularly among adolescents and older adults^11,12^. Previous research has also highlighted the importance of public support for successful policy implementation. Surveys conducted in the United States and Norway have reported high levels of approval for smoke-free environments, underscoring the role of societal engagement in tobacco control efforts^13-15^.

Despite this growing body of international evidence, research – particularly in the Chinese context – remains limited in its use of real-world public feedback data, such as unsolicited complaints, consultations, and inquiries, to evaluate behavioral responses and identify enforcement challenges following legislative changes. Such routinely collected data may provide complementary insights beyond traditional survey-based approaches.

The Shanghai 12320 Health Hotline functions as an accessible public reporting and consultation platform, systematically recording citizen feedback related to health policy implementation. Hotline data capture community concerns, perceived compliance, and enforcement challenges associated with smoke-free regulations. To address the aforementioned research gap, the present study investigates public responses to Shanghai’s smoking ban, which was initially enacted in 2011 and substantially strengthened in 2017. Using tobacco-related call records from the 12320 Health Hotline, we analyze public feedback surrounding the 2017 legislative amendment to identify major concerns and commonly reported violation settings. Specifically, this study seeks to address two research questions: 1) ‘What primary concerns are reflected in public hotline calls regarding the smoking ban?’ and 2) ‘How may these findings inform future monitoring and refinement of tobacco control policies?’.

## METHODS

### Study design and sample

This study adopted a text-mining-based data analysis design using anonymized call records obtained from the Shanghai 12320 Health Hotline^13^. The hotline is a government-operated public health service platform in Shanghai, China. Tobacco-related call sheets were collected between 1 March 2016 and 1 March 2018, with data access authorized for academic research by the relevant municipal health authorities. During this two-year period, a total of 41614 tobacco-related call records were generated and archived by the hotline system.

Importantly, identification of tobacco-related calls was embedded within the routine operational workflow of the hotline system. At the point of intake, operators initially triaged incoming calls and classified them as tobacco-related or non-tobacco-related. Calls identified as involving tobacco-related complaints or consultations were routinely transferred to a dedicated tobacco control team responsible for handling such inquiries. As a result, tobacco-related call records were systematically identified and documented at the source rather than selected retrospectively from a pre-existing archive.

To examine public responses following regulatory change, temporal profiling of call volumes was conducted across the entire study period (March 2016–March 2018). This analysis identified March 2017 – the first month after the smoking ban amendment was implemented – as the period with the highest volume of tobacco-related hotline activity. Based on this observed peak, the primary analytical window focused on call sheets recorded between 1 and 31 March 2017, to capture immediate patterns in public reporting and inquiry following the smoking ban amendment.

Each call record was assigned a unique identifier and contained standardized information fields, including call date and time, caller-reported locations, narrative descriptions of call content, processing actions taken, and response measures provided by hotline staff^13,16^. Only call sheets identified through the initial triage process as tobacco-related – namely, complaints, consultations, help-seeking requests, or suggestions – were eligible for inclusion. Calls unrelated to smoking, duplicate entries, or records with incomplete or insufficient information were excluded by design. All datasets were fully anonymized prior to analysis to ensure caller confidentiality.

Ethical approval for this study was granted by the Medical Ethics Review Committee of Shanghai Jiao Tong University School of Medicine (Approval No. SJUPN-201904).

### Data processing and text mining analysis

Within the identified peak period, a total of 6937 call sheets met the inclusion criteria and were included in the final analysis. Textual narratives were extracted and preprocessed prior to analysis.

Chinese-language text data underwent word segmentation to convert unstructured narrative content into analyzable lexical units. Natural Language Processing (NLP) techniques were subsequently applied to identify recurring themes and to extract information related to reported smoking violation settings.

Based on thematic features, calls were classified into five qualitative categories: complaints, consultations, help-seeking, suggestions, and uncategorized. Initial classification was conducted using automated NLP-based clustering. To enhance classification reliability, two researchers independently reviewed ambiguous or mixed-content entries and reached consensus through manual validation. This combined automated and researcher-validated workflow enabled standardized categorization of public feedback patterns using established text-mining approaches^17,18^.

### Statistical analysis

Descriptive statistical analyses were conducted to summarize call volumes, call types, and reported locations. Frequencies and percentages were calculated using Microsoft Excel 2019 (Microsoft Corporation, USA) and IBM SPSS Statistics version 26.0 (IBM Corp., Armonk, NY, USA). Inferential statistical testing was not performed, as the objective of the study was descriptive characterization of public feedback rather than hypothesis testing or causal inference.

## RESULTS

**Figure 1:**
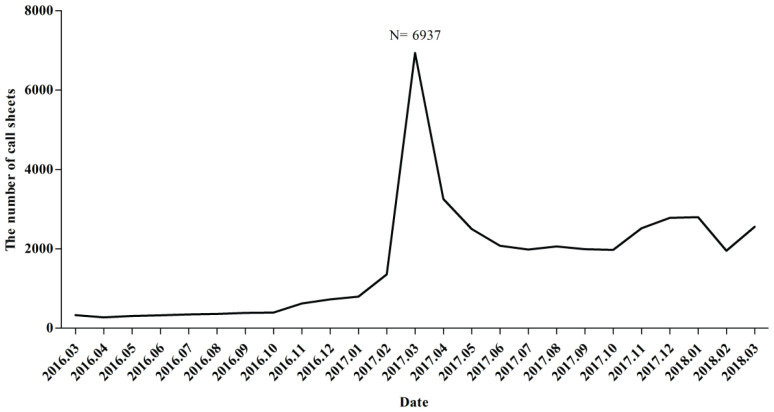
Monthly distribution of smoking-related call sheets to the 12320 Health Hotline following the implementation of the smoking ban, Shanghai, China, March 2016 – March 2018

Following the implementation of the smoking ban amendment on 1 March 2017, the number of calls to the 12320 Health Hotline peaked at 6937 records in March 2017 (Figure 1). This marked a significant increase in call volume compared to previous months. Despite a noticeable downward trend in the number of calls in the 12 months following the Amendment’s implementation, the volume of calls in March 2017 remained the highest observed during the study period. This suggests that the ban initially prompted heightened public awareness and engagement, likely driven by both curiosity and a desire to report violations.

### Types of call sheets

**Figure 2:**
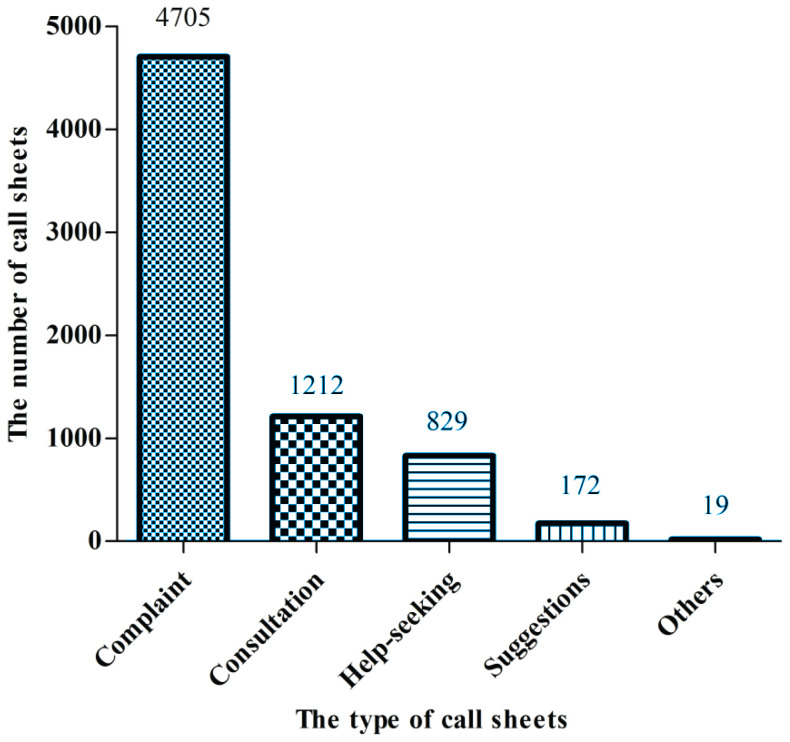
Distribution of tobacco-related call sheet types received by the 12320 Health Hotline in Shanghai, China, March 2017

Analysis of the ‘Descriptive Information’ field showed that call sheets were grouped into five distinct categories: complaints (n=4705; 49%), consultations (n=1212; 13%), help-seeking (n=829; 9%), suggestions (n=172; 2%), and uncategorized (n=19; <1%) (Figure 2). Complaints comprised nearly half of all call sheets collected during the study period, making them the most frequently reported type. Consultations ranked second, representing approximately one-eighth of all recorded calls. Help-seeking requests accounted for 9%, indicating that a notable proportion of callers sought practical assistance related to smoking-related concerns. Suggestions represented a smaller share (2%), while fewer than 1% of call sheets could not be categorized due to insufficient or unclear narrative content.

An examination of data specifically for March 2017, the month immediately following the implementation of the amended smoking control regulation, revealed a marked increase in complaint-type call sheets, which accounted for 68% of all calls in that month. This rise represented a notable proportion compared to the overall dataset. In comparison, consultations represented a smaller share relative to complaints during this same period.

A keyword search was performed within the consultation category to examine emerging concerns. The term ‘e-cigarette’ was identified in 184 call sheets, representing approximately 3% of all tobacco-related calls. This indicates that a subset of callers sought clarification or information related to electronic nicotine delivery systems. These mentions were mainly embedded within inquiries about regulatory coverage, enforcement boundaries, and the status of e-cigarettes under existing tobacco control laws.

### Locations mentioned in the call sheets were predominantly workplaces and hospitality service providers

**Figure 3:**
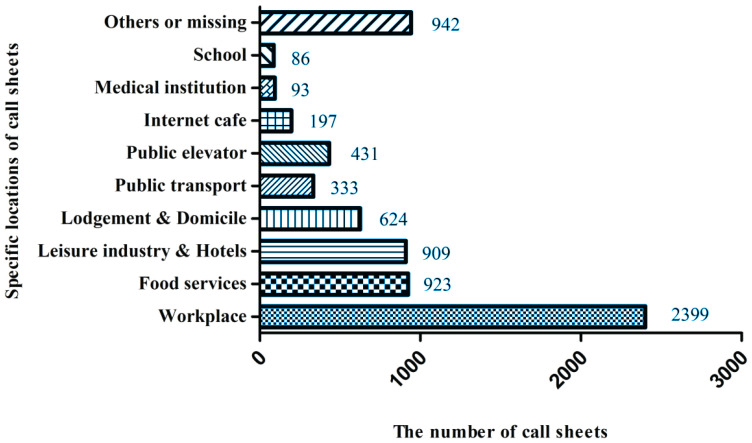
Reported violation locations mentioned in tobacco-related call sheets to the 12320 Health Hotline in Shanghai, China, March 2017

Categorization of reported locations was based on the ‘Location’ field within each call sheet. A total of 2399 mentions (35%) were associated with workplaces, making this the most frequently reported location type (Figure 3). Food and beverage establishments followed with 960 occurrences (14%), while leisure and hospitality venues accounted for 902 occurrences (13%). These location types collectively accounted for more than half of all reported call sheet entries. Calls referencing schools were the least frequent, recorded in 86 call sheets (1%). Other location types appeared less prominently and were grouped under miscellaneous categories.

Workplace-related calls included references to offices, administrative buildings, factories, and other professional environments where indoor smoking was reportedly observed. Food and beverage establishments included restaurants, coffee shops, bars, and small eateries. Leisure and hospitality-related locations comprised hotels, entertainment venues, fitness centers, and recreational clubs. Calls categorized under schools included references to both primary and secondary educational institutions.

## DISCUSSION

### The role of social supervision in tobacco control

The significant public response to tobacco control legislation – evidenced by the spike in hotline activity following implementation of the smoking ban amendment – illustrates the important role of social supervision in strengthening tobacco control. The high volume of calls demonstrates strong public interest and a willingness to participate in monitoring smoking violations^13,19^. However, the considerable number of inquiries and help-seeking calls indicates that many citizens lack a detailed understanding of the specific provisions of the smoking ban amendment. This finding highlights the need for more effective communication strategies and targeted public education efforts.

While the initial surge in calls was likely driven by pre-implementation promotional campaigns, the subsequent decline suggests that these campaigns may have had limited sustained impact. This pattern underscores the importance of continuous public education and ongoing engagement initiatives. Future campaigns should not only raise general awareness but also improve understanding of specific requirements under the smoking ban amendment, thereby fostering a more informed, proactive, and participatory population in support of tobacco control initiatives^18,19^.

### Targeted measures based on location

Analysis of the locations mentioned in the call sheets revealed that workplaces were the most frequently reported settings for smoking violations, consistent with findings from a 2002 smoking survey in China showing a high prevalence of smoking among working adults, who often smoke in groups^17^. In addition, complaints related to food and beverage establishments were common^20,21^, highlighting the need for stronger enforcement of the smoking ban amendment in these high-risk public venues. In contrast, schools were less frequently mentioned, which may reflect lower smoking prevalence, higher policy awareness, or more effective enforcement in educational settings.

These location-specific reporting patterns suggest that implementation of the smoking ban amendment could be strengthened by tailoring enforcement strategies to specific environments. Customizing enforcement efforts may improve compliance, particularly in settings where smoking remains culturally or socially embedded. Such targeted approaches can support more efficient and context-sensitive implementation of tobacco control policies.

### The need for e-cigarette regulation

A notable limitation of the smoking ban amendment was its initial exclusion of e-cigarettes, despite their increasing prevalence and growing public concern regarding regulation^22^. Electronic nicotine delivery systems often fall outside conventional tobacco control frameworks, creating regulatory ambiguity and enforcement challenges. International health authorities, including the World Health Organization, have emphasized the need to incorporate e-cigarettes into comprehensive tobacco control strategies to ensure policy coherence and population protection^23^. In China, these products did not clearly fall under existing pharmaceutical or tobacco product categories at the time, further complicating governance^24^.

In the present study, 184 consultation calls referenced e-cigarettes, accounting for approximately 3% of all calls, indicating increasing public concern about their use, health implications, and regulatory status. While some Chinese cities implemented early restrictions aligned with traditional tobacco control policies^25^, neither the national smoking ban amendment nor the Shanghai-specific regulations had included e-cigarettes at the time of data collection^26^. In October 2022, however, e-cigarettes were formally incorporated into both national and Shanghai smoking control regulations. This policy update represents a significant step toward a more comprehensive tobacco control framework and enhanced public health protection^27^.

### Supervision and fines in relation to the smoking ban amendment

In the context of the smoking ban amendment, many countries have adopted structured enforcement mechanisms and financial penalties to strengthen smoke-free legislation. For example, the UK Health Act of 2007 introduced fines for both individuals and businesses that fail to comply^28^. Spain and the United States similarly implemented smoke-free laws supported by penalty-based enforcement and public supervision mechanisms^14,29^. Several Southeast Asian countries, including Thailand, Singapore, and the Philippines, have also adopted smoke-free regulations reinforced by financial penalties^30^.

These international experiences suggest that the effectiveness of the smoking ban amendment may be closely linked to the clarity, visibility, and enforcement of penalties. In Shanghai, enforcement efforts have been strengthened since the implementation of the smoking ban amendment, including fines for violations in public buildings and transportation systems. Recent enforcement reports indicate increasing compliance and greater use of penalties against both individuals and businesses. These developments provide useful insights into how Shanghai may further refine enforcement strategies by drawing on international experience while adapting them to local contexts.

### Technological innovation: the 12320 Hotline and NLP analysis

The integration of the Shanghai 12320 Hotline with Natural Language Processing (NLP) techniques represents an innovative approach to monitoring public responses to the smoking ban amendment. The hotline facilitates direct communication between citizens and regulatory authorities, enabling individuals to report violations, seek clarification, or provide feedback^31^. Application of NLP methods allowed large volumes of unstructured call narratives to be systematically processed and transformed into interpretable, actionable data.

This approach provides insight into public perceptions, areas of misunderstanding, and enforcement challenges associated with the smoking ban amendment. By leveraging digital reporting platforms and analytical tools, policymakers may better identify emerging concerns, refine communication strategies, and strengthen tobacco control implementation. The methodological framework demonstrated in this study may also be applicable to other public health policy domains that rely on citizen engagement and real-time monitoring.

### Future research and policy implications

Future research should further examine the long-term effects of the smoking ban amendment on smoking behaviors, public awareness, and compliance using longitudinal approaches. Integrating multiple sources of citizen-generated data – such as health hotlines, digital reporting platforms, and social media – may strengthen real-time monitoring of policy implementation and emerging enforcement challenges.

Additional studies are needed to explore demographic and socioeconomic differences in reporting behavior and policy awareness. Comparative and mixed-methods designs may help identify underserved populations, clarify reporting motivations, and better understand barriers to effective enforcement.

From a policy perspective, tobacco control strategies should remain adaptive, including addressing new nicotine delivery products, strengthening enforcement across settings, and incorporating citizen-generated data into routine evaluation of the smoking ban amendment. Cross-sector collaboration among policymakers, public health agencies, researchers, and community organizations may further support sustainable tobacco control efforts.

### Limitations

Several limitations should be acknowledged. First, this study relied on analysis of hotline call records and therefore cannot establish causal relationships between the implementation of the smoking ban amendment and changes in smoking-related behaviors. In addition, due to data anonymization requirements, individual-level demographic information – such as age, gender, and socio-economic status – was unavailable, limiting examination of population-specific reporting patterns.

Second, hotline users may represent a subset of individuals who are more engaged or aware of the smoking ban amendment, which could introduce selection bias and limit generalizability. Although data cleaning procedures were applied, multiple calls related to the same incident or location may still have occurred, contributing to potential duplicate-reporting bias.

Finally, residual confounding cannot be ruled out. Variations in enforcement intensity, media exposure, public education activities, or local implementation practices over time and across settings may have influenced reporting behavior independently of the smoking ban amendment.

## CONCLUSIONS

This study examined public responses to the smoking ban amendment in Shanghai using hotline-based data and text-mining methods, highlighting the value of citizen-generated reports for monitoring policy implementation and public engagement. The findings demonstrate that hotline data can capture public reactions following implementation of the smoking ban amendment and provide actionable insights into location-specific enforcement challenges, as well as emerging regulatory issues such as e-cigarette use.

Although increased public participation reflects growing awareness of tobacco control, continued efforts are needed to enhance understanding of the smoking ban amendment and promote consistent enforcement across settings. Overall, the analytical framework applied in this study offers a scalable and transferable approach for assessing public engagement and implementation challenges in tobacco control and other public health policy domains.

## Data Availability

The data supporting this research are available from the authors on reasonable request.
